# The relationship between risk, the dark triad traits, and empathy

**DOI:** 10.1192/j.eurpsy.2021.1175

**Published:** 2021-08-13

**Authors:** T. Kornilova, E. Pavlova, Y. Krasavtseva

**Affiliations:** Department Of Psychology, Lomonosov Moscow State University, Moscow, Russian Federation

**Keywords:** Dark Triad Traits, risk, empathy, rationality

## Abstract

**Introduction:**

Empathy is generally viewed as a “positive” trait, while the Dark Triad traits are regarded as a “negative” side of a Dark personality. The perception of Risk is less univocal, as it plays a role in both courage and questionable behavior.

**Objectives:**

We posed the following research questions: 1. Is risk linked to empathy and the Dark Triad traits? 2. Which traits help distinguish between participants with contrasting latent profiles (determined cumulatively for the specified personality variables)?

**Methods:**

Participants (n=250) completed three questionnaires: the Dirty Dozen, Personality Factors of Decision-making and the Questionnaire of Cognitive and Affective Empathy (QCAE). Correlation and Latent profile analysis (LPA) were performed.

**Results:**

Risk was linked to Machiavellianism, psychopathy, and decentration (positively) and to emotion contagion and affective empathy (negatively). Rationality was positively correlated with cognitive empathy. Machiavellianism correlated negatively with rationality and online simulation (a cognitive empathy subscale). Empathy subscales were linked to psychopathy (negatively) and to narcissism (positively). LPA established two latent profiles: the smallest BIC value was obtained for the model with two profiles (log-likelihood: -3204.013, df=77, BIC=-6833; VEE). Analysis of means revealed that Class 1 was characterized by significantly higher Dark Triad values and higher Risk, whereas Class 2 was characterized by lower Dark Triad traits, lower Risk, and higher Rationality (see Figure 1).

**Figure:**
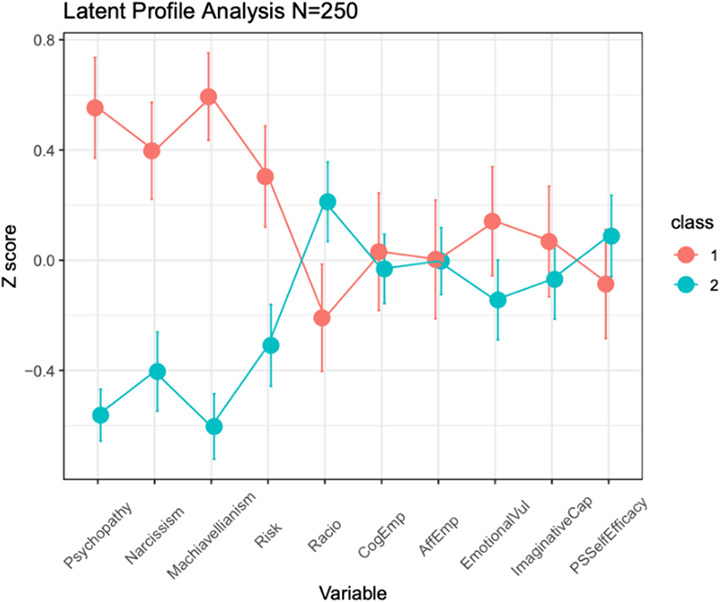

Figure 1. Latent profile analysis

**Conclusions:**

The Dark Triad traits and Risk are the more discriminative variables, while Empathy subscales do not help distinguish between the two classes of participants. The study was supported by the Russian Foundation for Basic Research, project 19-29-07069.

